# A diver-operated hyperspectral imaging and topographic surveying system for automated mapping of benthic habitats

**DOI:** 10.1038/s41598-017-07337-y

**Published:** 2017-08-02

**Authors:** Arjun Chennu, Paul Färber, Glenn De’ath, Dirk de Beer, Katharina E. Fabricius

**Affiliations:** 1 Max Planck Institute for Marine Microbiology, Bremen, Germany; 2 Australian Institute for Marine Science, Townsville, Australia

## Abstract

We developed a novel integrated technology for diver-operated surveying of shallow marine ecosystems. The HyperDiver system captures rich multifaceted data in each transect: hyperspectral and color imagery, topographic profiles, incident irradiance and water chemistry at a rate of 15–30 m^2^ per minute. From surveys in a coral reef following standard diver protocols, we show how the rich optical detail can be leveraged to generate photopigment abundance and benthic composition maps. We applied machine learning techniques, with a minor annotation effort (<2% of pixels), to automatically generate cm-scale benthic habitat maps of high taxonomic resolution and accuracy (93–97%). The ability to efficiently map benthic composition, photopigment densities and rugosity at reef scales is a compelling contribution to modernize reef monitoring. Seafloor-level hyperspectral images can be used for automated mapping, avoiding operator bias in the analysis and deliver the degree of detail necessary for standardized environmental monitoring. The technique can deliver fast, objective and economic reef survey results, making it a valuable tool for coastal managers and reef ecologists. Underwater hyperspectral surveying shares the vantage point of the high spatial and taxonomic resolution restricted to field surveys, with analytical techniques of remote sensing and provides targeted validation for aerial monitoring.

## Introduction

The need to efficiently monitor marine and freshwater ecosystems is now more urgent than ever for their effective conservation and resource management^1, 2^. Surveys of shallow benthic ecosystems require both fine spatial resolution, to capture the small size (cm–dm) and high diversity of organisms, and large areal coverage (1–100 km^2^) to adequately represent the vast extent of marine and freshwater habitats. Benthic survey and monitoring programs seek to quantify habitat composition (e.g. coverage of biota and abiotic substrata), spatial and geomorphological properties (e.g. depth, topography, rugosity, bathymetry), and physico-chemical parameters (e.g. light field, temperature, pH, oxygen) at the appropriate spatial and temporal scales^3^.

Coral reefs are the most biologically diverse marine ecosystems on the planet, valued at 30 billion dollars in economic returns annually, facing significant deterioration due to a nexus of local (e.g. eutrophication, sedimentation, pollution) and global (ocean acidification, global warming, sea level rise) stressors^4–8^. Surveying and identifying reef benthos is a particularly challenging task given their extreme taxonomic diversity and spatial complexity.

Traditionally, reef surveys have required expert ecologists to perform *in-situ* surveys by identifying and counting taxa underwater. While direct visual surveys provide accurate observations, they can only cover a limited reef area under typical logistical constraints. They require extensive field time, are affected by the biases of the ecologist’s expertise, and do not generate a lasting record of the habitat structure for re-analysis. Recognition of the necessity for both great taxonomic resolution and large spatial extent of surveys^9, 10^, has highlighted the need for new and integrated technologies to efficiently inform spatial analyses and management of coral reef ecosystems.

The advent of cheap imaging technology allows to complement direct observations with image-based surveys. Images are collected either underwater (0.1–5 mm pixels) or from aerial platforms (10–1000 cm pixels)^11, 12^. The advantages of image-based surveys are the creation of a lasting record of the habitat, and decoupling the acquisition and analysis time of surveys. Modern reef monitoring is most commonly based on underwater photographic surveys, which enable relatively fast surveys over large reef areas (hundreds of m^2^). Throughput has further increased through the use of autonomous underwater vehicles (covering many thousands of m^2^)^13, 14^. The resulting large data sets^15^ have shifted the bottleneck of generating results to the analysis of large collections of survey images to identify reef substrata/organisms^16, 17^. Such analysis typically entails sub-sampling the high-resolution images and visual identification by experts to estimate benthic coverage^18, 19^. Recent developments have improved algorithmic automation and structuring of the annotation effort, through projects such as CATAMI and CoralNet, to alleviate the backlog of field survey analysis^20–23^. Other efforts have explored the use of spectral filtering to estimate benthic coverage^24, 25^.

Modern reef monitoring programs also include Earth observation platforms (planes or satellites) to rapidly cover vast areas of reefs (covering 10–1000 of km^2^)^26^. The extensive spatial scale and high temporal resolution of Earth observation platforms enables the simultaneous study of spatial and temporal patterns over large reef areas^27^. Remote sensing imagery is typically collected using multispectral imagers with effective pixel dimensions of 1–100 m. The analysis of remote imagery is challenging^28^ as the size of the pixels are far greater than most reef organisms. This leads to ‘mixed pixels’ with convolved spectral signatures of disparate benthic targets^29^, which limits mapping precision and necessitates specialized deconvolution analysis^30, 31^. The complex optical path between imager and target through effects of the atmosphere, clouds, sunlight, water surface and depth also confounds the benthic signatures^32, 33^. Irrespective of the spatial scale of the measurements, the accuracy of image-based benthic mapping depends on the availability of high quality ground-validation data^3, 10, 34^.

One imaging technology of high potential is hyperspectral imaging, which involves the collection of hundreds of spatial pixels each with an optical spectrum resolved into hundreds of contiguous wavelength bands. The spatially resolved and dense collection of high-resolution spectra allows a better estimation of the inherent structure and variability in the detailed spectral signature of a target. The spectral features that allow health assessment or discrimination between benthic biota are typically constrained in narrow spectral regions, arising due to arise due to the interaction of light with the types and densities of functional molecules, such as photopigments and chromoproteins, in the tissue of the organisms^35^. Hyperspectral sensors have the spectral resolution to resolve such small spectral features for benthic targets^28, 36^. The conserved association of biological pigments to spectral features extends the *in-situ* mapping capabilities of hyperspectral data to microbial communities^37–39^, and allows assessing the physiological status of organisms and communities^40–42^. The correspondence between photopigments and coral hyperspectral reflectance has shown to be consistent across biogeographic regions^43, 44^. The collection of *in-situ* spectral reflectances towards a global library of benthic signatures is considered an important step towards the validation and improvement of remote reef monitoring^10, 45^.

We developed a novel instrument called HyperDiver, which is a diver-operable hyperspectral, topographic and physico-chemical surveying system for underwater habitats. Based on field studies in a tropical coral reef, we developed survey protocols to simultaneously capture data about benthic coverage, topography, irradiance and water chemistry along transects in a rapid and efficient manner. We present data from a transect (color-accurate images, rugosity metrics, photopigment maps, and detailed habitat composition maps) to demonstrate the versatile benefits of underwater hyperspectral imaging for surveying shallow marine habitats. The applications of such a method promise benefits for both field-based and remote monitoring of coral reefs and other shallow marine and freshwater habitats.

## Materials and Methods

### Instrumentation

**Figure 1:**
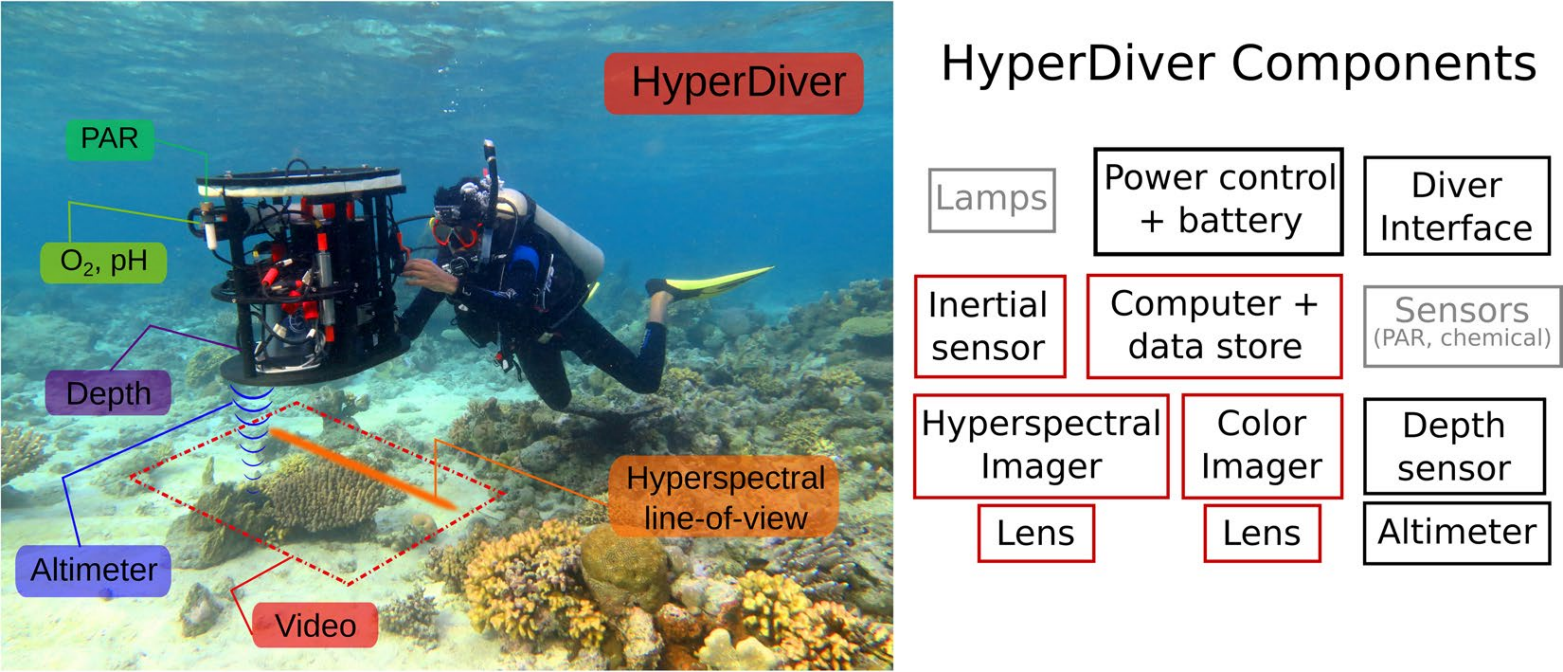
The HyperDiver system operated by a SCUBA diver to survey a shallow coral reef. The system simultaneously captures hyperspectral and color images, bottom altitude, depth, irradiance and chemical parameters. The field-of-view of the imagers depends on the altitude from the bottom, and the hyperspectral images can deliver centimeter-scale spatial resolution. The neutrally buoyant system can be easily navigated along transects following standard survey sampling protocols. The schematic shows the components of the system, with the instruments inside the main cylinder in red and optional components in gray. Details of the weight and cost of the system are in the supplement. (Photo by Sam Noonan).

The HyperDiver system (Fig. 1) was designed to maximize the information gathered from diver-based underwater surveys of shallow marine habitats. The goal was to simultaneously capture high-resolution color and hyperspectral images and topographic profiles of the benthos, together with light and physico-chemical parameters of the water column. The main electronics cylinder contains a push-broom hyperspectral imager (Pika 2, Resonon Inc.) with a spectral range of 400–900 nm sampled at ~1.5 nm resolution (480 fixed bands, 640 spatial pixels), and a color imager (Blackfly, Point Grey Inc.) with three channels in each pixel (1900 × 1200). The imagers are fitted with custom-built electronically-controlled objective lenses, that allow adjustment of the zoom, focal length and aperture. A single-board computer is included for operational control and data storage. The imagers are placed at the bottom of the cylinder and capture light through a clear acryl optical window. The imagers are positioned such that the hyperspectral line-of-view lies within the field-of-view (1–1.5 m wide) of the color imager.

HyperDiver also includes a suite of other sensors. A light sensor (LI-192, LI-COR Inc.) is attached at the top of the system to measure downwelling photosynthetically active radiation (PAR). At the bottom of the instrument is an acoustic altimeter (Micron Echosounder, Tritech Intl. Ltd.) to measure the altitude of the imagers from the seafloor. Navigational information is captured by an inertial measurement unit (HMC6343, Honeywell Inc) and a pressure gauge (CTE9003, First Sensor AG) to record orientation, acceleration, compass heading and water depth. A set of chemical loggers (RBR duo, RBR Ltd.) measures the pH, dissolved oxygen and redox potential of the water column. HyperDiver can also include downward facing halogen lamps to provide additional broadband illumination for operation under low-light conditions, such as at depth or night time. The central operational node is a computer that runs custom control software, which allows modular and programmable control of the various components of the system^39^. Interactive underwater operation by the diver is enabled through a custom electronic module using magnetic switches and a display.

HyperDiver draws power from light-weight 12 V battery packs. The power rating of the battery is 15 ampere-hours. With a normal current consumption of 1.5 A, the on-board batteries allow operation for 10 hours. Operation time with 120 W halogen lamps is about 1.5 hours. The batteries are rechargeable to full capacity within 2 hours.

HyperDiver is designed for remote field operations and transport. The system provides a communication port so that data can be downloaded or settings changed without opening the underwater housing, thus significantly reducing the operational time for re-servicing. The whole system fits in two suitcases and can be dis- and reassembled on site with simple mechanical tools within 1–2 hours. The position of the various subsystems is configurable. It is rated for operation to a water depth of 50 m. The weight of the system in air is ~32 kg, and can be easily adjusted to be neutrally buoyant in water with a balanced horizontal orientation.

### Survey operation

The typical considerations for planning underwater imaging surveys, such as depth, cloud cover, water state, direction, etc, also apply while planning HyperDiver transects. Although imaging surveys of benthos do not strictly require it, measuring tapes are typically employed to mark the distance and orientation of the transects. Once a transect is selected, a gray reference board (25 × 25 cm) is placed on the reef bottom at one or both ends of the transect. Once the diver initiates data acquisition using the switch interface, data from all sensors and imagers are logged independently with timestamps and metadata to the system’s on-board storage. During the dive all sensors except the imagers are active, and during the scan the imagers are activated as well. The diver swims along the transect pushing the neutrally buoyant instrument, with the imagers facing the benthos. At the end of the transect, the operator ends the data acquisition through another switch, and can proceed to the next transect.

The scanning protocol conforms more with video rather than photographic surveys. This is because the hyperspectral imager acquires spectrally resolved images of a line-of-view, and successive acquisitions of lines while moving along the transect are compiled into a hyperspectral transect image. The length, width, velocity and sampling rate of the line-of-view determine the spatial resolution and range of the transect image. The length and width are determined by the altitude and the focus of the front optics, whereas the velocity and frame-rate determine the distance between consecutive acquisitions. The length of the line-of-view is internally resolved by the imager into 640 pixels (the lateral resolution) and the distance between lines and their width (determined by frame rate and speed of swimming) determines the transverse resolution. Smooth linear trajectories over the transect area help to avoid sidewards motion of the imager, which can distort the image coherence.

Maintaining strict control on scanning velocity and altitude in a reef can be challenging due to complex topography and water conditions, but the diver should aim to limit variations in imaging parameters within the transect. To aid with this, the user interface displays real-time parameters such as altitude, heading, imager saturation, etc throughout the dive. Scanning transects at an altitude of 1 m and swimming speed of 20 m/min samples a line-of-view of 1–1.5 m length every 1–2 centimeters. This results in pixels of 0.2 × 2 cm and a data rate of about 10 MB/s, adding up to about 1.5 GB of hyperspectral data for a 50 m transect, collected in <3 min. These data rates are lower than those of high definition video used for reef surveys, but higher than single snapshot images.

## Analysis and Results

### In-water handling

HyperDiver was employed to conduct *in-situ* surveys of coral reefs on Normanby and Dobu Islands in the Milne Bay Province of Papua New Guinea. The sites are known to contain a rich biodiversity of coral reef taxa^46, 47^. Our surveys focused on near-shore and outer reefs covering a variety of benthic types including corals, macroalgae, turf algae, coral rubble, seagrass, sponges, anemones, etc.

The HyperDiver system was easy to handle underwater, with similar ergonomic handling to other underwater video systems (Fig. 1). No specialized skills were necessary different from using other underwater imaging systems. By following the indications in the user interface, a diver is able to safely navigate, position and record data with HyperDiver in order to perform benthic surveys. Our surveys were conducted along transects in shallow water (<8 m depth) under ambient sunlight. With weak currents, it was possible to survey the benthos at 15–20 meters per minute. With a hyperspectral line width of 1–1.5 m, we covered 15–30 m^2^ per minute with a pixel resolution of 1–2 cm in the spectral image. Working with a buddy diver, 20–25 transects of 25 m (500–650 m^2^) could be performed efficiently within the no-decompression time limits of one dive, allowing for a coverage of up to 2000 m^2^ per day, assuming three shallow dives. Over a period of 3 weeks, we captured ~400 survey transects from the region.

### Transect Analysis

**Figure 2:**
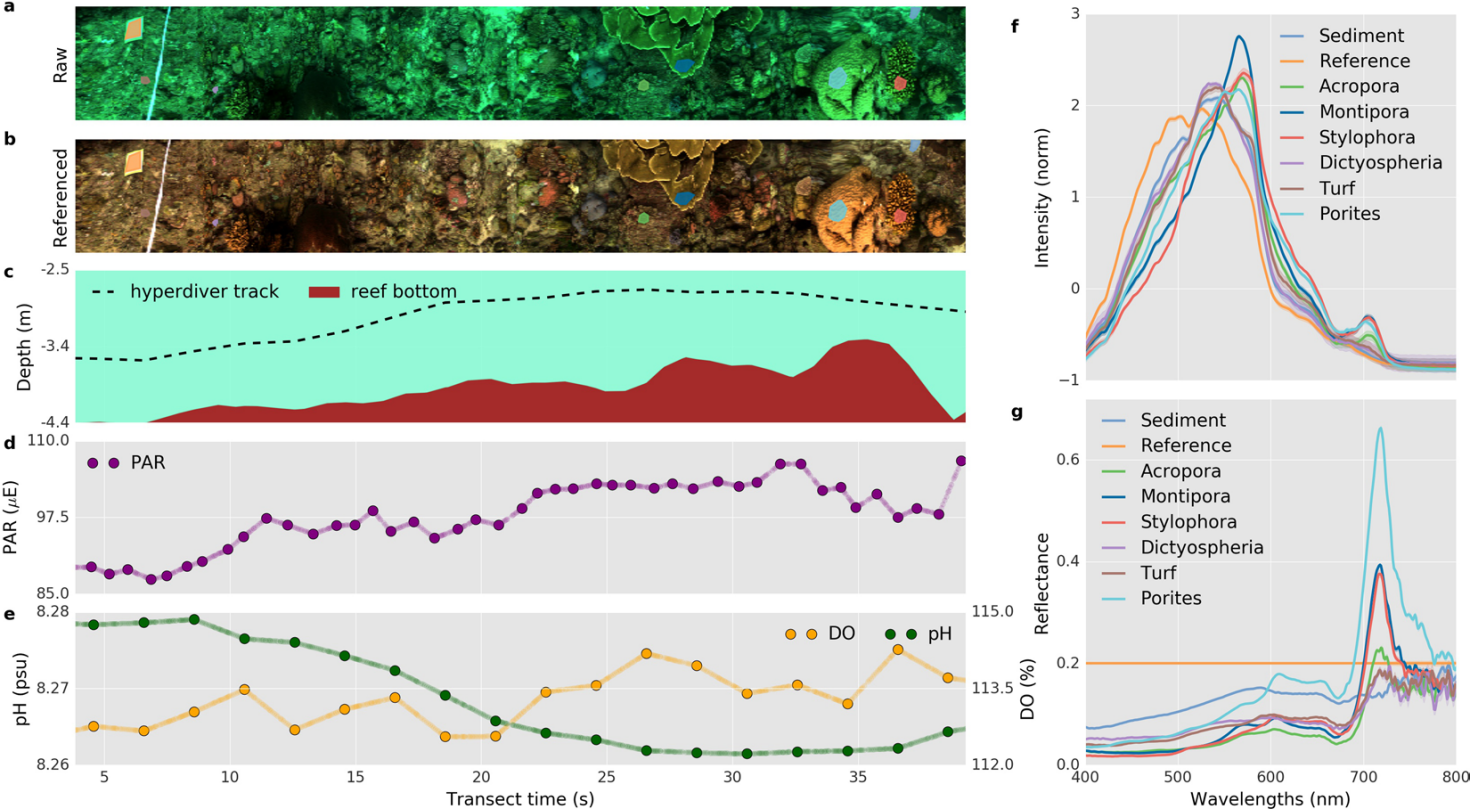
A HyperDiver survey transect delivers a multi-faceted dataset. (**a**) The measured scene visualized as a 3-channel color image derived from the hyperspectral data. (**b**) The same scene after reference-correction of spectra (plastic board, top left in scene) provides a true-color view of the benthos. (**c**) Depth and altitude information in combination generate a topographic profile of the bottom along the transect, useful to calculate rugosity. Additional sensors measured the (**d**) PAR intensity, (**e**) dissolved oxygen and pH over the transect. The transect scene covered ~16 m^2^ in ~35 seconds with 1–2 cm resolution. Regions of interest (colored polygons in A and B) were selected in the scene to derive the average spectral intensity (**f**) and reflectance (**g**) of specific benthic targets.

Each transect with the HyperDiver system captures spectral images, depth, altitude, irradiance, and chemical profiles of O_2_ & pH. One such transect is shown in Fig. 2 as an example.

Color images of the transect (Fig. 2a) can be derived from the spectral image by plotting the pixels corresponding to the wavelengths in the blue (460 nm), green (540 nm) and red (640 nm) parts of the spectral range. Generated images were stretched to rectify proportions for visualization. The image revealed a blue-green tinted view of the scene, due to the preferential absorption of longer-wavelengths by seawater. We applied a white-balance correction by using the reflectance from the gray board to generate a true-color image (Fig. 2b), which removed the tint and provided a more color-accurate representation of the reef floor. The use of an *in-situ* reference works reasonably well for the assumption that the average light field does not significantly change over the course of the transect (1–2 min). The spatial resolution of the image was sufficiently high to localize the various benthic targets. Various targets in the transect area, marked by polygons in the true-color views (Fig. 2b), could therefore be investigated for their uncorrected spectra (Fig. 2f) or corrected reflectance spectral signatures (Fig. 2g).

The additional sensors on HyperDiver collected complementary data, further characterizing habitat properties along the transects. Depth and altitude information, measured by a single-beam acoustic altimeter and captured at 1 Hz, were used to localize the position of HyperDiver in the water column and map the topographic profile of the seafloor (Fig. 2c). The topographic profile data allows us to calculate the arc-chord rugosity index (ratio of contoured to planar distance) of the surveyed area^48^, which is an important measure for the habitat complexity of a reef. The downwelling PAR intensity (Fig. 2d) and the O_2_ and pH profiles (Fig. 2e) were also captured along the transect. With a measurement of the water’s transmissivity, the PAR irradiance reaching the reef benthos can also be calculated (not shown). Overall, a single HyperDiver transect efficiently captures a comprehensive set of survey parameters regarding benthic composition, reef morphology, PAR and the physico-chemical environment.

### Spectrometric mapping

Hyperspectral images capture and resolve a much greater proportion (hundreds of bands) of the light field compared to standard color images (three bands). The availability of a contiguous spectral bands at each pixel allows the application of various spectrometric techniques to create several abundance or discriminant maps from the same spectral image.

**Figure 3:**
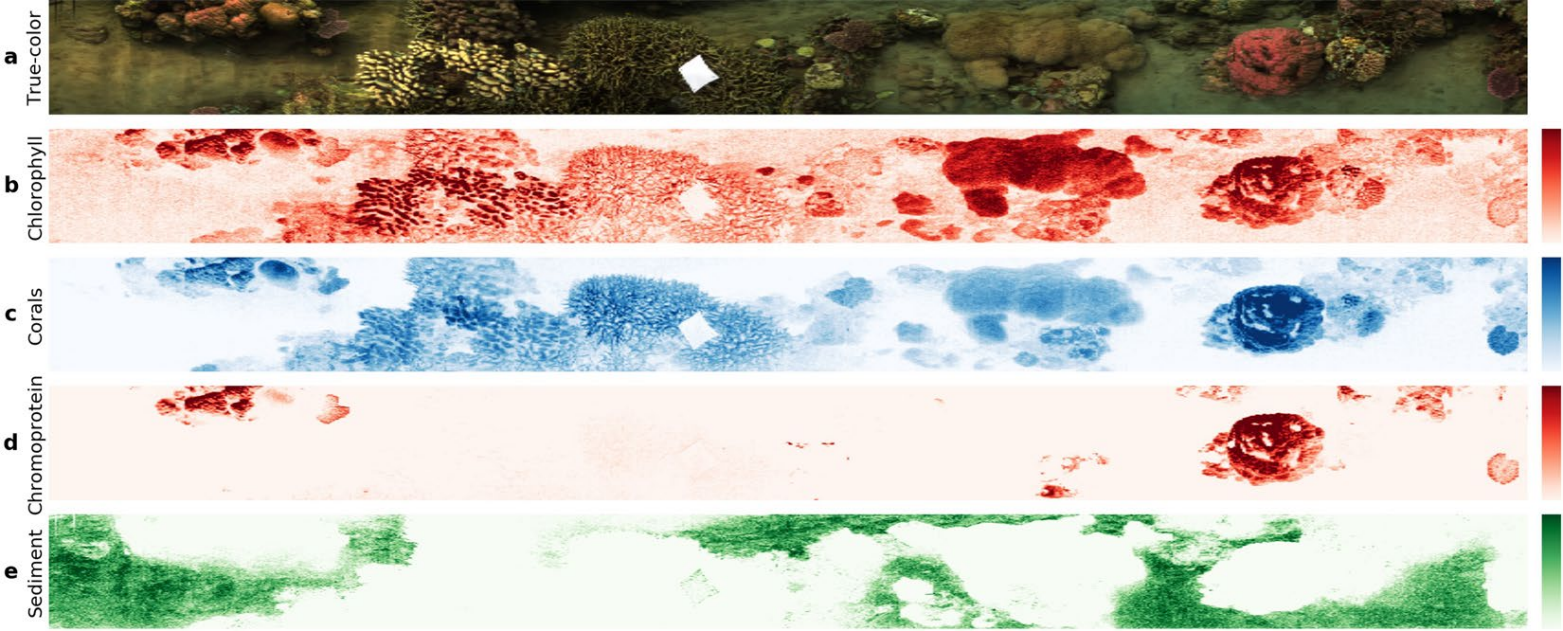
Semi-quantitative spectrometric maps generated from the transect scene shown in (**a**). Derivative analysis of the reflectance spectra was used to create abundance/discriminant maps of chorophyllic photopigments (**b**), coral substrata (**c**), coral chromoproteins (**d**) and sediments (phycoerythrin) (**e**). Color intensity reflects the value of the calculated spectrometric indices for the targets.

The true-color map shown in Fig. 3a was derived from the hyperspectral image as described before. The spectra of the various benthic targets in the image were inspected to identify any specific associated signatures. Spectral signatures can be quantified through various means, such as ratiometric indices or derivative analysis^39, 49^. Specifically, the second derivative of the spectra in each pixel was calculated at the wavelength of maximum *in-vivo* absorption of chlorophyllic pigments (670 nm). This value, mapped in Fig. 3b, can be interpreted as the (uncalibrated) concentration of chlorophyll at each pixel location^50^.

Earlier work on spectral reflectance of corals have identified a characteristic strong positive slope at the infrared shoulder of the chlorophyllic absorption which discriminated coral from sand and algae^51, 52^. The positive values of the first derivative calculated at 700 nm in the spectra of each pixel were mapped in Fig. 3c, which indicated good discrimination of the coral substrata. Additionally, corals host a range of chromoproteins in their tissue that exhibit specific spectral features from the ultra-violet to the red^35^. We identified an absorption feature at 580 nm in spectra associated with certain coral colonies. By mapping the second derivative at this wavelength (Fig. 3d), we found this signal to be consistent across the surfaces of certain species of stony corals, including massive *Porites* and *Pocillopora* colonies. The identity of the chromoprotein is unconfirmed, but considering the spectral characteristics, it is likely to be the previously described ‘green-yellow protein’^53^.

Similarly, we identified an absorption feature at 605 nm in some sediment pixels of the image and mapped the second derivative values at this wavelength (Fig. 3e). This method clearly discriminated the sedimentary regions of the transect, and is likely due to the absorption of phycoerythrin, which is an accessory photopigment found in cyanobacterial and chryophyte microalgae^54^.

Although the various spectrometric maps provided good discrimination for their targets, the quantitative aspect of these maps requires further analyses to validate and calibrate the sources of the spectral signatures^35^. However, these abundance maps can provide useful guidance in the field to choose study sites, design sampling strategies, and reduce sample wastage for eco-physiology studies in reefs.

### Categorical mapping

Supervised classification is a technique to decompose an image and assign each pixel a semantic category corresponding to its identity or type (eg. coral, sediment, algae). Supervised classification entails two phases of operation. In the training phase, a statistical model is generated based on a subset of the data which associates particular pixel groups with their designated categorical identities. In the prediction phase, the trained model is used to predict the categorical identities of the pixels of the entire image, thus generating a classified map. The quality of the classifier model is typically validated by comparing the predicted category against the known category for a set of pixels that were not used in the training phase.

**Figure 4:**
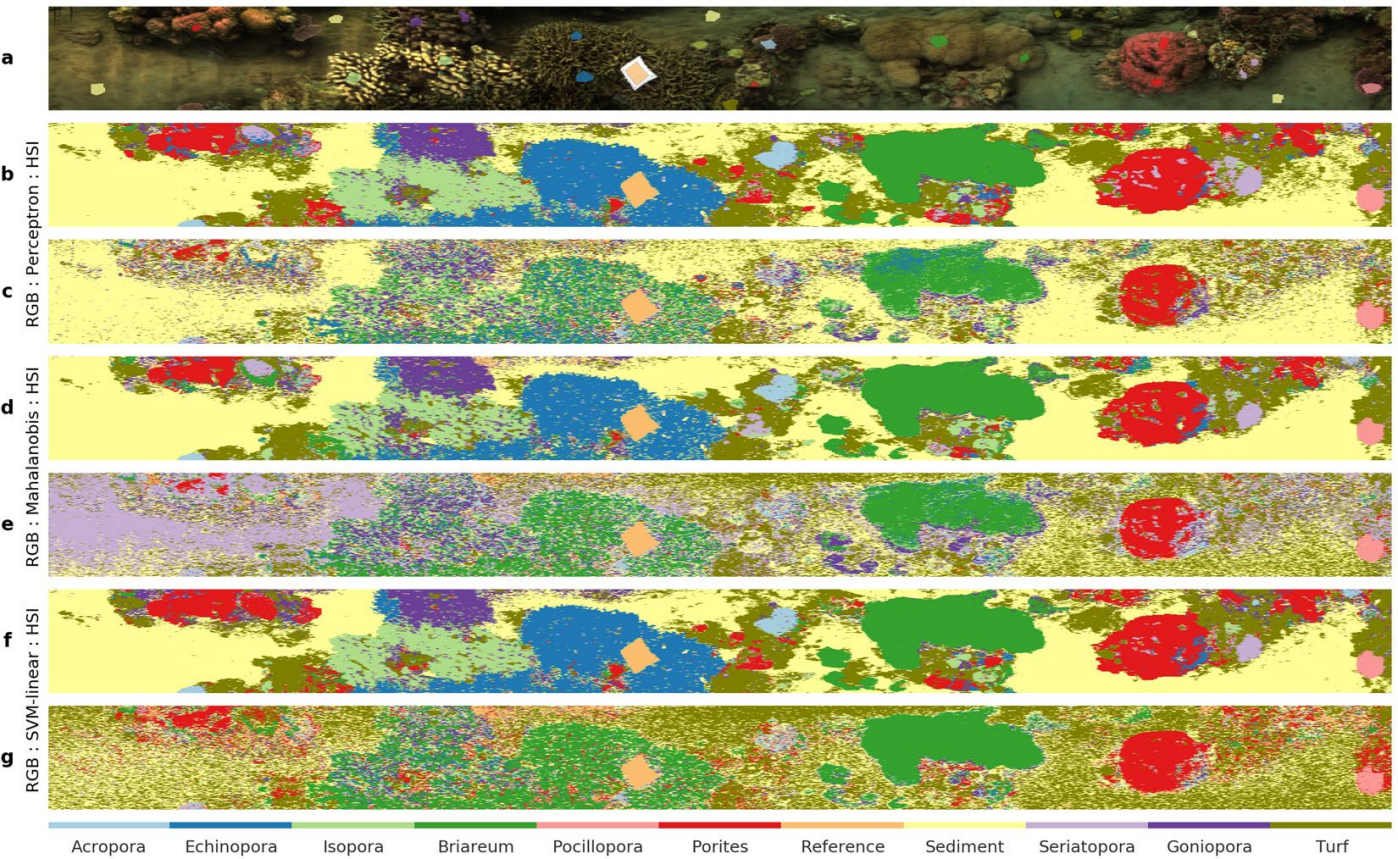
Classified benthic maps generated from supervised classification of the transect scene shown in (**a**). The classifier models were trained on the 1.3% of the total image pixels annotated for 11 benthic classes (see colored polygons in (**a**) and colormap legend). The classified maps were generated for hyperspectral and color RGB pixels separately using the classification algorithms Multi-layer Perceptron (**b**,**c** respectively), Mahalanobis distance criterion (**d**,**e** respectively) and Support Vector Machines with linear basis function (**f**,**g** respectively). The performance metrics of the classifiers are shown in Fig. 5.

Supervised classification of the pixels of the test transect image were used to compare the performance when operating on pixels of hyperspectral resolution (“HSI”) versus the three-channel color (“RGB”) pixels. The HSI pixels used Z-normed spectral intensities of the 324 bands in the wavelength range 400–750 nm (discarding the infrared region with no useful signal), and the RGB pixels used the intensity values for the red, green and blue bands. We created an annotated dataset through visual inspection of the test transect. Specifically, small polygons were created in the image and each polygon section was annotated with the identity of the benthic target it contained (Fig. 4a). We aimed for broad coverage of the transect scene and identified 11 categories as training targets: *Acropora*, *Briareum*, *Echinopora*, *Goniopora*, *Isopora*, *Pocillopora*, *Porites*, *Reference*, *Sediment*, *Seriatopora* and *Turf* (algae). The benefits of supervised classification is most pronounced when accurate classification can be achieved with a small training dataset. In our case, the annotated dataset for all sections only contained 1.8% of the total pixels, of which 75% (1.3% of total) was used for training and the remaining 25% (0.5% of total) was used for validation of the classifier model.

We applied three algorithms to create separate classifier models: Multi-layer Perceptron (a type of artificial neural network), Mahalanobis distance, and Support Vector Machines (SVM). These algorithms have been used in various hyperspectral classification efforts, and a discussion of their details is beyond the scope of this text, but can be found in the domain literature^55–57^. All algorithms were applied separately to the HSI and RGB pixels of the same training dataset to create classifier models for each pixel type. The generated classifier models were evaluated by comparing their accuracy, precision and recall. Precision, also known as producer accuracy, is a measure of the ability of the classifier to correctly label pixels to the right class, i.e. the ratio true positives:(true positives + false positives), while recall (user accuracy) is its ability to identify the pixels of a certain class, i.e. the ratio true positives:(true positives + false negatives).

**Figure 5:**
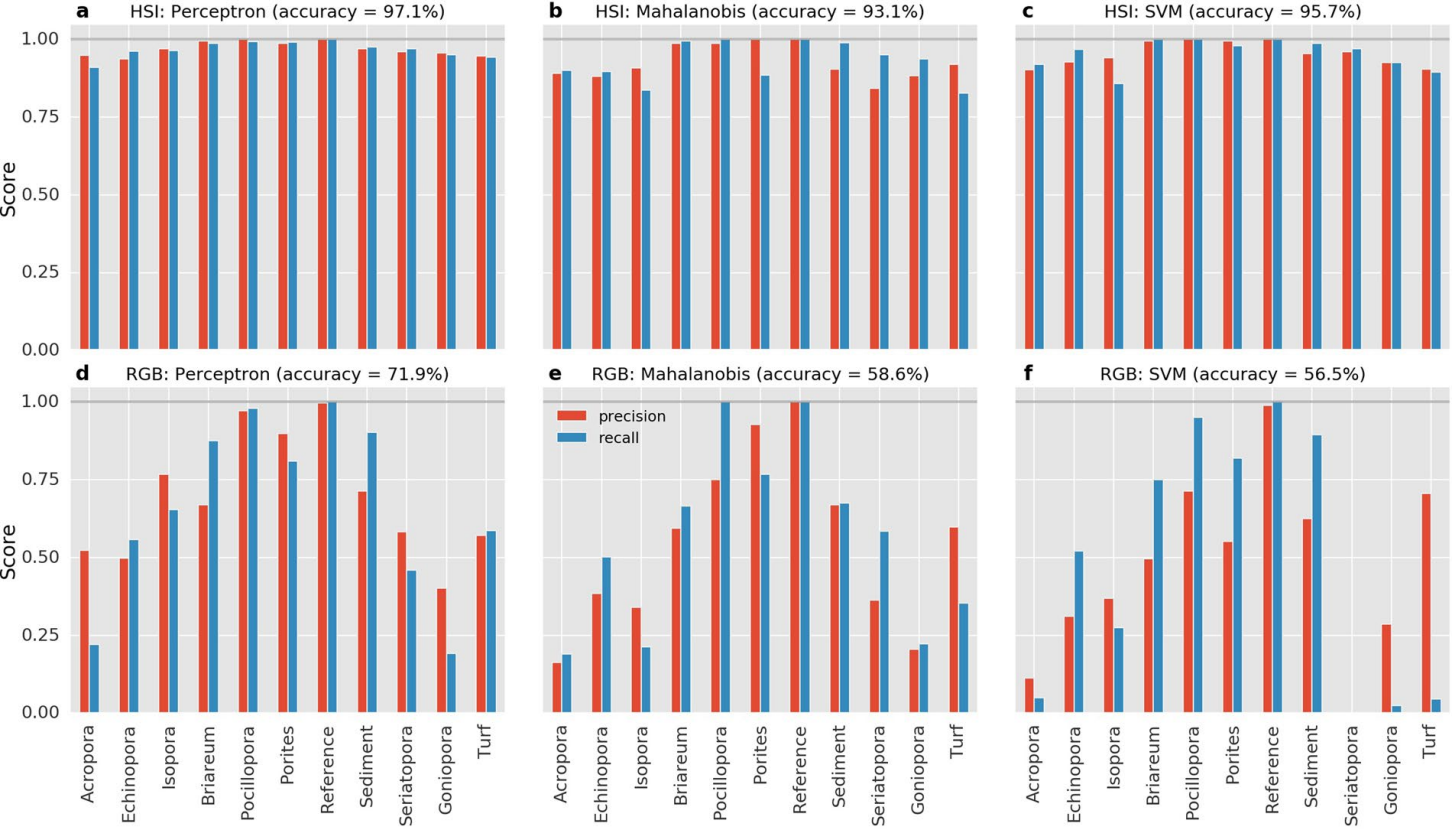
Performance of classifier models trained on annotated pixels (1.3% of total) from the test transect image (see Fig. 4a) were evaluated through metrics of precision, recall and overall accuracy. Metric scores are shown for classifiers trained separately on HSI (panels a–c) and RGB (panels d–f) pixels using three different algorithms (see panel text). Precision (also called producer accuracy) measures the ability of the classifier to avoid false positives and recall (also called user accuracy) is the ability to find all samples of a particular class.

Of the three algorithms, the Perceptron algorithm showed the best performance on both HSI and RGB pixels. Validation of the classifiers revealed that the accuracies of those trained on HSI pixels were 93–97%, whereas those trained on the color pixels were 57–72% (Fig. 5). For all categories and algorithms, the HSI classifiers outperformed the RGB classifiers (Fig. 5 panels a–c vs d–f). The RGB classifiers performed poorly particularly in identifying branching coral categories, suggesting the utility of high spectral resolution for reef substrata with complex macro-optic structures.

Each classifier was then applied to classify the HSI and RGB pixels of the entire transect image, and were mapped with the same ordered colormap (Fig. 4b–g). Overall, the classified maps showed a strong structural resemblance to the transect scene shown in the true-color image (Fig. 4a). The high degree of similarity between the classified maps from the different algorithms suggest that the information in HSI pixels contained sufficient detail for discriminating between the target classes. It is evident that the HSI classifiers (panels b,d,f) outperformed the RGB classifiers (panels c, e.g.) in capturing the benthic composition of the transect. A few aspects of the classified maps are worth noting. The HSI classifiers accurately mapped the areas of the various coral colonies and sediment regions with clear boundaries, whereas the RGB classifiers suffered from confusion and were unable to correctly map some of the coral colonies (compare *Briareum*, *Echinopora* and *Isopora*). For the HSI classifiers, the recognition of coral colonies (e.g. *Porites* in panel b) occurred across the transect scene, even when training data was marked only at a few sparse locations. All HSI classifiers were able to correctly recognize the different sediment regions including those relatively shadowed (top edge of scene), whereas the RGB classified maps exhibit fine-scale noise in these regions. Interestingly, even though the small set of training pixels for *Turf* algae were only marked on overgrown coral colonies, the HSI classifiers categorized regions across the scene as *Turf*, including in various sediment regions. This is understandable due to the similar phototrophic microbial communities in turf algae and sedimentary microalgae. The Mahalanobis distance classifier showed the greatest disparity between the HSI and RGB cases (panels d and e). This stems from the algorithm being designed for large dimensional data, for which the three-band RGB pixels are a poor fit.

The HSI classifier models were able to effectively generalize the structure in the hyperspectral radiance from a very small amount of training data and perform accurate discrimination between 11 categories spanning from sediment to coral genera. Of particular note is the ability to map the coverage of turf algae, which are difficult to quantify visually, and branching coral colonies with complex macro-optic structure. Overall, these analyses show that underwater hyperspectral images of coral reefs are very effective for mapping benthic habitat structure with a high degree of taxonomic resolution. The scalability of this approach to all transects from a multi-reef survey remains to be assessed, and will be the focus of a subsequent study.

### Effect of altitude

**Figure 6:**
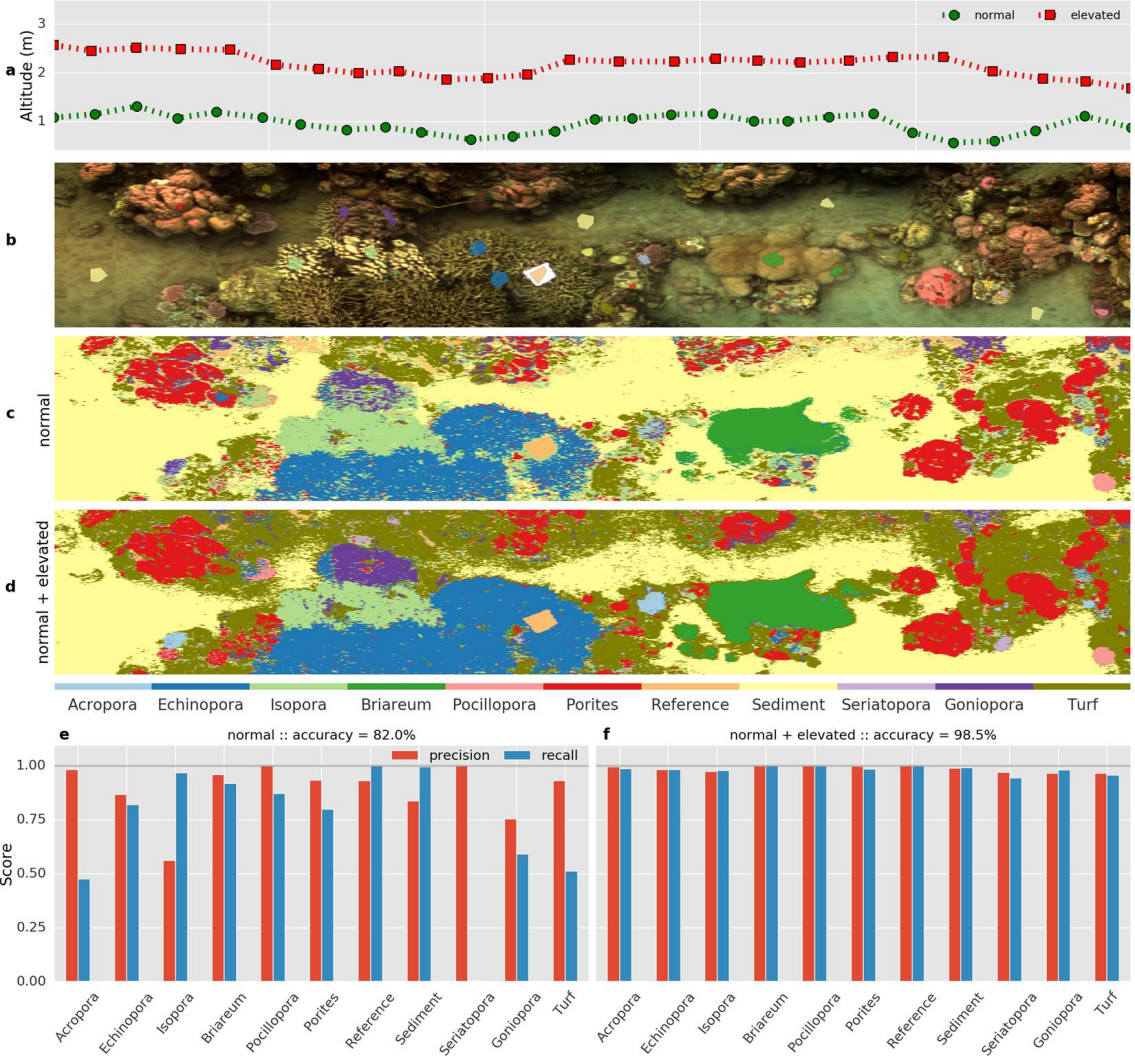
Effect of the (underwater) altitude during measurement of the transect was compared by capturing the same transect area at ‘normal’ and ‘elevated’ altitudes (panel a). The normal altitude image is shown in Fig. 4a, and the elevated altitude image in panel (b). Multi-layer perceptron classifiers were used to create classified benthic maps of the elevated altitude image. The map in panel (c) was created by a classifier trained on the annotated pixels from the normal altitude image only, whereas the map in panel d was created from a classifier trained on annotated pixels in both altitude images (panel b and Fig. 4a). The performance metrics for the two classifiers are shown in panels (e and f) respectively.

The optical environment and target distance from which hyperspectral images are acquired influences the quality and range of the spectral data. Seawater, although generally clear in coral reefs, exhibits preferential absorption and dispersion of the light passing through it. To understand the effect of surveying altitude on the quality of the acquired spectral images, we re-scanned the transect area captured from a “normal” altitude of about ~1 m again from an “elevated” altitude of ~2 m (Fig. 6a). The elevated trajectory provided a wider view of the transect area (compare Figs 4a and 6b). Visual identification of the various targets identified from the normal transect was possible also in the elevated transect image. Due to the nature of compiling an underwater hyperspectral image (see *Survey Operation*), these two transect images exhibit some minor variations in the spatial structure captured, such as a small region of motion-derived ‘smearing’ near the branched corals (Fig. 6b).

To assess the effect on the spectral quality, we created polygon sections on the elevated altitude image (colored regions in Fig. 6b) at about the same locations for the same benthic categories as in the normal altitude image (regions in Fig. 4a). We compared the mean and standard deviation of the spectra for each category between the two altitude images (see supplement). We found that the general structure of the spectral features remained the same, however with different degrees of variability within and between categories. For example, the variability in the spectra for *Porites* and *Isopora* was larger within the image at normal altitude than the difference in the (average) spectra between the normal and elevated images. On the other hand, for the *Turf* category, the difference between the average spectra between the normal and elevated images was larger than the variability within either image.

We investigated in how far the classifier models could analyze the two altitude images correctly. The Perceptron classifier trained only on annotations from the normal altitude image (Figs 5a and 4a,b) was used to classify the HSI pixels in the elevated altitude image. The resulting classified map (Fig. 6c) showed a strong resemblance in structure (spatial locations and categories) to the transect scene. We evaluated the classification performance by inspecting the predictions of the classifier on the annotations in the elevated image to determine accuracy, precision and recall. We found that the overall accuracy in classifying the elevated image was lower at 82% compared with 97% for the image at normal altitude (Fig. 6e vs Fig. 5a). Specifically, in the elevated image, some categories were predicted with reduced precision (*Echinopora*, *Isopora*, *Sediment* and *Turf*), while others were predicted with reduced recall (*Acropora*, *Echinopora*, *Seriatopora*, *Goniopora* and *Turf*). These confusions correspond with the degree of inherent variability in the category spectra and the similarity between categories (see supplement). Overall, it appeared that the categories with heterogeneous macro-optic structure such as branching corals faced a greater loss in classification accuracy at elevated altitude.

As a further step, we investigated whether we could improve the classification performance by combining annotations from the spectral images captured at different altitudes and spatial resolutions. As in the case of the normal altitude image, the annotation effort for the elevated image was low at 1.5% of the pixels (Fig. 6b). The annotated pixels from the two images were combined and 75% were used to train a new classifier with the Perceptron algorithm. The classifier performance was validated against the remaining 25% and found to be 98% accurate (Fig. 6f). The resulting classified map (Fig. 6d) reflects this improved performance, such as in the correctly classified regions of *Goniopora*, *Seriatopora* and *Porites* categories. The regions categorized as *Sediment* and *Turf* show larger divergence between the two classified maps (panel c,d), possibly due to the natural overlap between sedimentary and turf micro-algal communities (compare spectra in supplement). The location and identities of the major coral categories, both massive and branching, were correctly marked by the classifier trained on images from both altitudes.

Overall, we conclude that the protocols for underwater hyperspectral surveying can have some leeway for choosing measuring altitude without significant loss of classification power, although some categories (like branching coral) are more affected than other more homogeneous categories. Within a 1–2 m range of altitude, the change in spectral quality was minimal and the loss in classification power could be regained through pooling the sparse annotations of different images together. Further efforts are necessary to evaluate the performance of classifiers trained on a large number of underwater hyperspectral survey images.

## Discussion

Our study demonstrates that underwater hyperspectral imaging provides significant and direct benefits for coral reef surveys. The hyperspectral reflectance of various benthic targets contains several signatures that are useful to assess taxonomic identity or physiological status^35^. Capturing this information across large reef areas enables analyses that can provide rich descriptions of reef habitats in a scalable and non-invasive manner. These benefits suggest that, given the costs and efforts involved in underwater surveys (see below), collection of underwater hyperspectral imagery will represent a significant improvement in the informational throughput of reef survey efforts without significant disruption of existing monitoring schemes.

HyperDiver packages several technologies that combines the modalities of remote sensing and field-based surveys, and is the first system to our knowledge to enable diver-operable underwater hyperspectral imaging and topographic surveying of shallow marine habitats such as coral reefs. The system was practical and efficient to survey large reef areas with a methodology that aligns well with existing techniques such as line or grid visual, photo or video transects. Surveys made with this method deliver information-rich observations of the benthic habitat by simultaneously recording visual and hyperspectral imagery, depth, topography, photosynthetic irradiance and seawater chemistry. The topographic profiles can be used to estimate the rugosity of the surveyed reefs, which is a useful metric for various ecosystem aspects such as habitat complexity, coastal protection, and a predictor for fish and macro-invertebrate assemblages^47, 58, 59^.

Although the centimeter-scale spatial resolution of the hyperspectral images is lower than the millimeter-scale resolution of modern color imagers, it was sufficient to identify and isolate the spectral reflectance at the organism level across the transect area. On the other hand, the high spectral resolution of hyperspectral images can be leveraged for versatile analysis of various ecological metrics of the habitat. Applying standard spectrometric analyses for specific spectral signatures can be used to create quantitative abundance maps (Fig. 3). The same spectral image can therefore produce estimations of the abundance of photopigments (e.g. chlorophyll, peridinin), coral chromoproteins, etc. Chlorophyll maps capture the photosynthetic potential of a whole section of reef benthos in a scalable fashion, and could even resolve the variations in abundance within the different sections of a coral head. This approach can also be scaled down to focus on particular targets simply by scanning closer or adapting the front optics of the imager. In the reef context, such chlorophyll abundance data (assessed on the transect scale, or separately for specific taxonomic groups) can provide important information on reef health. For example, it can be used to cost-effectively quantify coral pigment loss due to heat stress (coral bleaching), or increases in pigmentation in response to poor water quality^60–62^. Although not attempted in this study, the spectral metrics of chromophore abundance can be calibrated by chemical extractions of samples using laboratory techniques such as chromatography. This would enable scaling up the estimations of absolute abundances, while alleviating the effort for laboratory work and destructive sampling^42^. Another option is the application of previously developed bio-optical models for estimating photopigment abundances in specific target groups^42, 63, 64^. The use of optical filters and specialized illumination can be used for fluorescence-based analysis.

Benthic habitat structure, in terms of composition and coverage, can be derived by adopting statistical tools to classify the pixels based on their spectral properties. This approach seeks to create categorical maps for semantically structured habitat maps (Figs 4 and 6). The algorithms used in classification employ statistical models that perform better when a larger number of independent features are provided for training, hence capturing the inherent variability within and between the target categories. Color pixels contain only 3 channels of information, and as such the classifiers suffer confusion between disparate target groups with similar hue and brightness (Fig. 5). Hyperspectral pixels with hundreds of channels of information (spectral bands) capture the small but numerous differences in reflectance of the target groups (see Fig. 2f,g, supplement), which arise from their variable composition of photopigments, chromophores and optical structures (such as tissue, sediment matrix, etc). Our analyses show that hyperspectral imagery is conducive to automated mapping of the reef benthos with a high degree of taxonomic detail, while requiring very little manual annotation. The annotation effort can be further reduced by adopting techniques from recent efforts in computer vision research^23^.

Another rich avenue of development is the incorporation of localized spatial information of adjacent image pixels, which captures textural details like morphology and surface structures, to perform spatial-spectral classification^65^. Several modern algorithms, such as convolutional neural networks, seek to emulate the parallelism in human visual recognition by including multi-scale spatial pattern analysis in spectral images^66, 67^. This approach, called object-based image analysis, provides state of the art performance when a corpus of training data is available, and has been recently applied on hyperspectral images^68^. Therefore, utilizing semi-supervised and spectral-spatial classification methods would further improve the performance of classifying hyperspectral images of habitat surveys^65, 69^.

Apart from the direct benefit of efficient and accurate habitat mapping that hyperspectral surveys provide, this technique has the potential to significantly aid large-scale remote sensing efforts in monitoring coral reefs. Previous work suggests that the spectral reflectances of dominant coral families are conserved across geographic regions^43, 52^, primarily linked to their genetic lineage^42^. Generalization of the knowledge gained from successful classification studies are crucial to inform large-scale remote sensing efforts, but have generally suffered due to the inconsistencies and scalability of employed methods^35^. A primary limitation is the availability of sufficient high-quality ground-truthing data^3^. Underwater hyperspectral imaging, as enabled by the HyperDiver system, has the potential to fill this important gap. Imagery obtained from aerial or satellite imagers unavoidably include the confounding effects of complex optical path through the water column, the air-water interface and the atmosphere, which significantly deteriorates the information content useful for discriminating benthic biota. The seafloor-level hyperspectral images captured by HyperDiver contain dense spatially-coherent spectrally-pure pixels from various benthic targets (Figs 2 and 4), and obviate complex corrections of the effects of water and air columns. Additionally, the high-resolution pure spectra are useful for the unmixing analysis necessary in aerial images due to larger pixels which span disparate benthic targets. The efficient generation of validated high-resolution habitat maps represents the state of the art ground-truthing available for remote sensing, and additionally provides useful input to improve physical modelling of the optical effects of the water column. This capability also aligns well with the growing interest in ‘data fusion’, which attempts to combine data captured at different resolutions from different sources to create an ensemble model for classifying reef benthos^70, 71^.

Continued use of underwater hyperspectral imaging promises some novel benefits for field-based studies, such as those constrained to land or lab-based approaches^72, 73^. The improved taxonomic detail and spatial coverage in habitat description is of direct relevance to many ecological studies, and enables the assessment of hard to assess categories (such as turf algae or small coral colonies), and their pigment densities, in a seamless manner.

Improving some aspects of the HyperDiver system design would improve the cost-benefit advantage of adopting such technology. The presented HyperDiver system and methodology is easy to use by non-expert divers without biological training and requires no change to standard diver-based survey protocols. Given that the system can be assembled with simple tools, it does not add any significant overhead to time in the field. As such, the operational costs are similar to those outlined for other techniques^10^. The cost of HyperDiver’s components is around 22,000 EUR (see supplement), and expected to reduce for future instrument models. The described system is voluminous and would require some miniaturization to enable integration on unmanned platforms such as shallow water AUVs, and operate in more turbulent waters and on vertical shelves. Improvements in the capability to effectively georeference transects in shallow water settings would be useful to align field survey data for ingestion into remote sensing analyses. Compared to the analysis of color images, an increased computational load must be accounted for. The software tools^74^ necessary for such analysis are available for free (see supplement), and can be run on regular desktop computers. The ability to automate the mapping of benthic composition while retaining taxonomic detail and accuracy represents a major cost-saving, since a major component of the cost in scaling up reef monitoring is the salary cost required for analysis of survey photos^10^. The rich discriminatory ability of the presented algorithms for hyperspectral images allows to keep the annotation effort low while using entire transects instead of sub-sampled points for generating benthic composition maps.

Overall, underwater hyperspectral surveying is a novel contribution to the description of complex marine habitats such as coral reefs, and can efficiently deliver comprehensive maps of the benthic habitat space and their ecological and physiological properties. The technique combines the vantage point of field-based surveys and remote sensing, and can help foster the much-needed dialogue between field ecologist and remote sensing communities. Further development and application of such techniques will improve our ability to generate rich and timely information about the structure and status of marine habitats, such as coral reefs, which are urgently needed for coastal management efforts.

### Data availability

The datasets generated during and/or analyzed during the current study are available from the corresponding author on reasonable request.

### Ethics statement

All work was carried out ethically and within appropriate permissions received.

## Electronic supplementary material

Supplementary Information
