# Supplementary material for: A diver-operated hyperspectral imaging and topographic surveying system for automated mapping of benthic habitats
Source: Sci Rep. 2017 Aug 2;7:7122. doi: 10.1038/s41598-017-07337-y (PMC5541065; doi:10.1038/s41598-017-07337-y)
Supplement: Supplementary file 1 — Supplementary Information [file 41598_2017_7337_MOESM1_ESM.pdf]

Supplementary Information

for

A diver-operated hyperspectral imaging and topographic surveying system for automated mapping of benthic habitats

Authors:
Arjun Chennu, Paul Färber, Glenn De'ath, Dirk de Beer, Katharina E. Fabricius

A listing of the major components of the HyperDiver system, along with their approximate cost and weight (in air).

Component	Cost (€)	Weight (kg)
Hyperspectral imager	7500	
Color imager	500	
Computer + storage	800	13
Lens	700	
Inertial sensor	40	
PAR sensor	2200	0.9
Altimeter	2800	0.5
Depth sensor	200	0.4
Battery	370	4.2
Diver interface	300	0.9
Mechanical parts	2500	10.2
Underwater cables, connectors and ports	4000	1.4
**Total**	21910	31.5

Supervised classification

Details and parameters of the algorithms used for supervised classification are provided here:

- Multi-layer Perceptron: 1 hidden layers with 30 neurons, logistic activation function, L2 penalty parameter 0.1, maximum iterations 2000.
- Support Vector Machine: linear kernel, squared hinge loss function, penalty parameter 1 with inverse proportional scaling with class frequency in training samples.
- Mahalanobis Distance classifier: distance threshold 0

Effect of altitude

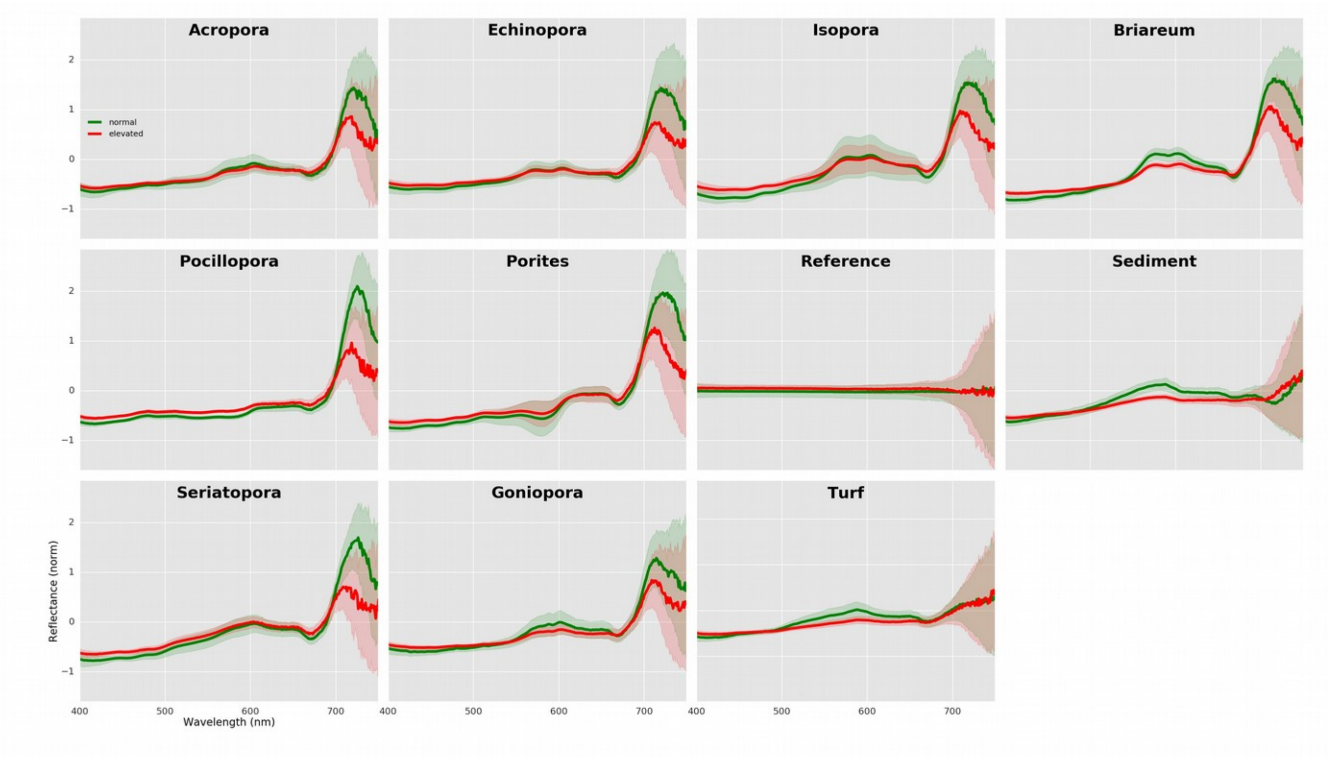

Figure 1: Comparison of the spectral properties of each category (see Figure 6 of main text) of the same transect scene measured from normal (1 m) and elevated (2 m) altitudes. The thick lines are the average Z-normed spectra of the class pixels, and the shaded bands are the standard deviations. Observe the generally conserved location of spectral features in both altitude images. The systematically large variability in the near infrared (>700 nm) is an artifact of division of near-zero values to calculate reflectance. In other spectral regions, the within image variability (thickness of band) can be higher than the between image variability (difference between means), such as for Isopora, Porites, Echinopora and Acropora. Sediment and Turf, which have similar spectral features, indicate different mean reflectances at the two altitudes. This is either a systematic annotation error, or a confoundment of categories.

Free software tools useful for analysis

- numpy www.numpy.org
- scikit-learn www.scikit-learn.org
- Spectral www.spectralpython.net
- scipy www.scipy.org
